# Transcriptomics of the Bed Bug (*Cimex lectularius)*

**DOI:** 10.1371/journal.pone.0016336

**Published:** 2011-01-19

**Authors:** Xiaodong Bai, Praveen Mamidala, Swapna P. Rajarapu, Susan C. Jones, Omprakash Mittapalli

**Affiliations:** 1 Department of Entomology, Ohio Agricultural and Research Development Center, The Ohio State University, Wooster, Ohio, United States of America; 2 Department of Entomology, The Ohio State University, Columbus, Ohio, United States of America; AgroParisTech, France

## Abstract

**Background:**

Bed bugs (*Cimex lectularius*) are blood-feeding insects poised to become one of the major pests in households throughout the United States. Resistance of *C. lectularius* to insecticides/pesticides is one factor thought to be involved in its sudden resurgence. Despite its high-impact status, scant knowledge exists at the genomic level for *C. lectularius*. Hence, we subjected the *C. lectularius* transcriptome to 454 pyrosequencing in order to identify potential genes involved in pesticide resistance.

**Methodology and Principal Findings:**

Using 454 pyrosequencing, we obtained a total of 216,419 reads with 79,596,412 bp, which were assembled into 35,646 expressed sequence tags (3902 contigs and 31744 singletons). Nearly 85.9% of the *C. lectularius* sequences showed similarity to insect sequences, but 44.8% of the deduced proteins of *C. lectularius* did not show similarity with sequences in the GenBank non-redundant database. KEGG analysis revealed putative members of several detoxification pathways involved in pesticide resistance. Lamprin domains, Protein Kinase domains, Protein Tyrosine Kinase domains and cytochrome P450 domains were among the top Pfam domains predicted for the *C. lectularius* sequences. An initial assessment of putative defense genes, including a cytochrome P450 and a glutathione-S-transferase (GST), revealed high transcript levels for the cytochrome P450 (CYP9) in pesticide-exposed versus pesticide-susceptible *C. lectularius* populations. A significant number of single nucleotide polymorphisms (296) and microsatellite loci (370) were predicted in the *C. lectularius* sequences. Furthermore, 59 putative sequences of *Wolbachia* were retrieved from the database.

**Conclusions:**

To our knowledge this is the first study to elucidate the genetic makeup of *C. lectularius.* This pyrosequencing effort provides clues to the identification of potential detoxification genes involved in pesticide resistance of *C. lectularius* and lays the foundation for future functional genomics studies.

## Introduction

"*Good night, sleep tight, don't let the bed bugs bite!*" This common nighttime verse now has become a precautionary catch phrase around the globe. Bed bugs (*Cimex lectularius* L.) are flightless, nocturnal, obligate blood-feeding ectoparasites that preferentially feed on humans. Bed bug infestations pose grave economic concerns and quality-of-life issues for households [1]. The resurgence of bed bugs poses an urgent situation as infestations are rampant globally, nationally, and locally. The control of these medicinally important insect pests in urban environments costs billions of dollars annually and typically requires the use of large quantities of pesticides/insecticides.

Individuals that are allergic to *C. lectularius* bites often experience itching and erythematous or papular urticaria-like dermatitis, which favors secondary infections like impetigo, ecthyma and lymphanigites [2]–[7]. *C. lectularius* infestations also result in anxiety, insomnia or worsening of an existing mental health condition [7]–[9]. However, the risk of transmission of human disease by *C. lectularius* is still not clear [10]. These ectoparasites are an important public health issue affecting all socioeconomic classes.

The association of *C. lectularius* and humans dates back to 1350 B.C. or earlier, as evidenced by well-preserved bed bug remains recovered from the Workmen's Village at el-Amarna, Egypt [11]. Bed bugs are not native to North America but rather were introduced by the early colonists in the 17^th^ century. *C. lectularius* were extremely common pests in the United States prior to World War II, however extensive use of dichloro-diphenyl-trichloroethane (DDT) and other long-lasting residual insecticides greatly reduced their numbers [12].

During the past decade or so, the resurgence of *C. lectularius* has been recorded across the globe including North America, Europe, Australia, and Eastern Asia with an estimated 100–500% annual increase in bed bug populations [13]–[17]. Survey by the National Pest Management Association and the U.S. Environmental Protection Agency (EPA) indicated that *C. lectularius* stress calls increased 81% during the last decade; the majority of bed bug complaints came from occupants of multi-unit apartment complexes. Furthermore, 76% of pest management companies confirmed that *C. lectularius* were the most difficult pest to control (www.pestworld.org). Several hypotheses have been proposed to explain the sudden resurgence of *C. lectularius* worldwide which include, but are not limited to, frequent international travel (to/from areas where *C. lectularius* remained common), increased exchange of used furniture, a shift from usage of broad-spectrum insecticides to more specific/selective control tactics such as baits for other urban pests, and insecticide resistance within the insect [18]–[25].

Resistance to pyrethroids (e.g., deltamethrin and lambda-cyhalothrin) appears to be widespread within U.S. populations of *C. lectularius* [23]. Pesticide resistance in *C. lectularius* is purported to result from point mutations in the open reading frames of voltage-sensitive sodium channel genes compared to pesticide susceptible populations [26]. However, the role of cytochrome P450s and glutathione S-transferases (GSTs) has yet to be established in pesticide resistance of *C. lectularius*. In many insects, both cytochrome P450s and GSTs have been shown to metabolize synthetic chemicals (insecticides/pesticides) and host plant allelochemicals [27]–[31]. The cytochrome P450 and GST detoxification systems catalyze physiological reactions that modify toxic compounds into water-soluble, non-toxic compounds that are excreted by insects.

Despite the high-impact status of *C. lectularius*, very little is known about this blood-feeding insect at the molecular level.The next generation sequencing methods (Roche 454, Solexa/Illumina, etc.) provide a unique opportunity for genomic exploration in non-model insect species wherein little or no molecular knowledge is available [32]. In particular, 454-sequencing technology based on the pyrosequencing principle has recently enabled the application of functional genomics to a broad range of insect species including *Melitaea cinxia* [33], *Zygaena filipendulae* [34], *Chyrsomela tremulae* [35], *Aphis glycines* [36]; *Manduca sexta* [37], [38], *Laodelphax striatellus* [39], *Stomoxys calcitrans* [40], *Dermacentor variabilis* [41], *Erynnis propertius and Papilio zelicaon* [42], and *Agrilus planipennis* [43]. In the current study we applied 454 technology to build a sufficiently large expressed sequence tag (EST) database for *C. lectularius*. Our results will allow for a better understanding of the physiology-driven molecular processes in *C. lectularius* and the identification of candidate genes potentially involved in insecticide resistance.

## Results and Discussion

### Transcriptomic analysis

**Figure 1:**
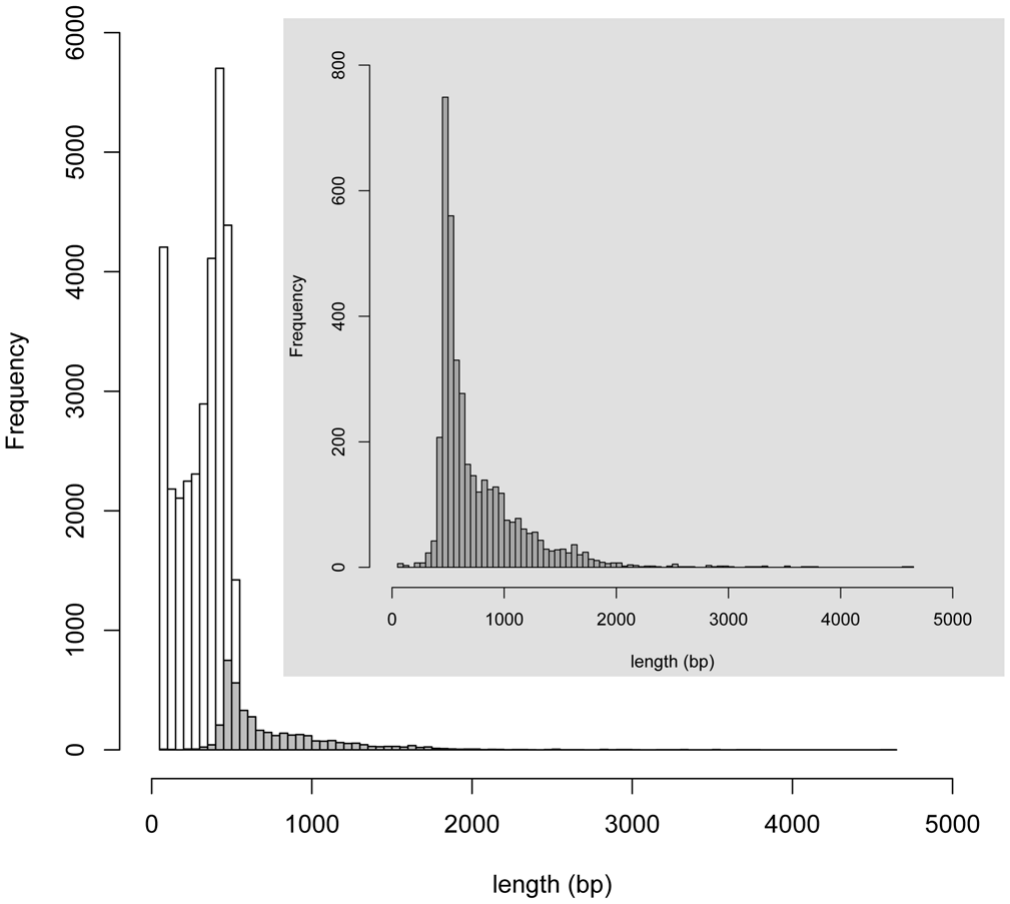
Summary of *Cimex lectularius* transcriptomic sequences. The contig sequences are represented by shaded bars and the singleton sequences by clear bars.

**Figure 2:**
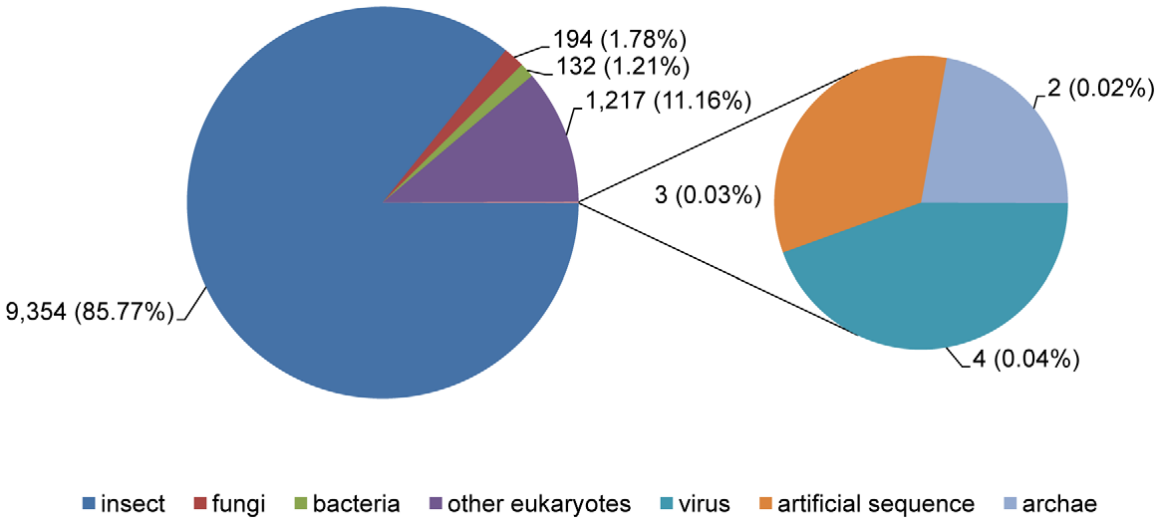
A pie chart showing species distribution of the top BLAST hits of the *Cimex lectularius* sequences to various insect species.

Roche 454 pyrosequencing of adult *C. lectularius* yielded a total of 216,419 transcriptomic reads with 79,596,412 bp, which were assembled into 35,646 ESTs (3,902 contigs and 31,744 singletons) (Figure 1) using the Roche Newbler program. The length of the contigs varied from 60–4,615 bp with an average contig length of 759 bp and totaling 2,962,366 bp. The singletons ranged from 50–863 bp with an average length of 313 bp and totaling 9,919,703 bp. From the current *C. lectularius* transcriptomic database, 29.6% transcripts showed significant similarity (E value <1e^−5^) to proteins in the GenBank nr database. As expected, the majority of the sequences (85.9%) were matched to insect proteins and the remaining were matched to non-insect eukaryotes (11.16%), fungi (1.78%), bacteria (1.21%), viruses (0.04%), Archaea (0.02% sequences) and artificial sequences (0.03% sequences) (Figure 2).

### Comparative analysis

**Figure 3:**
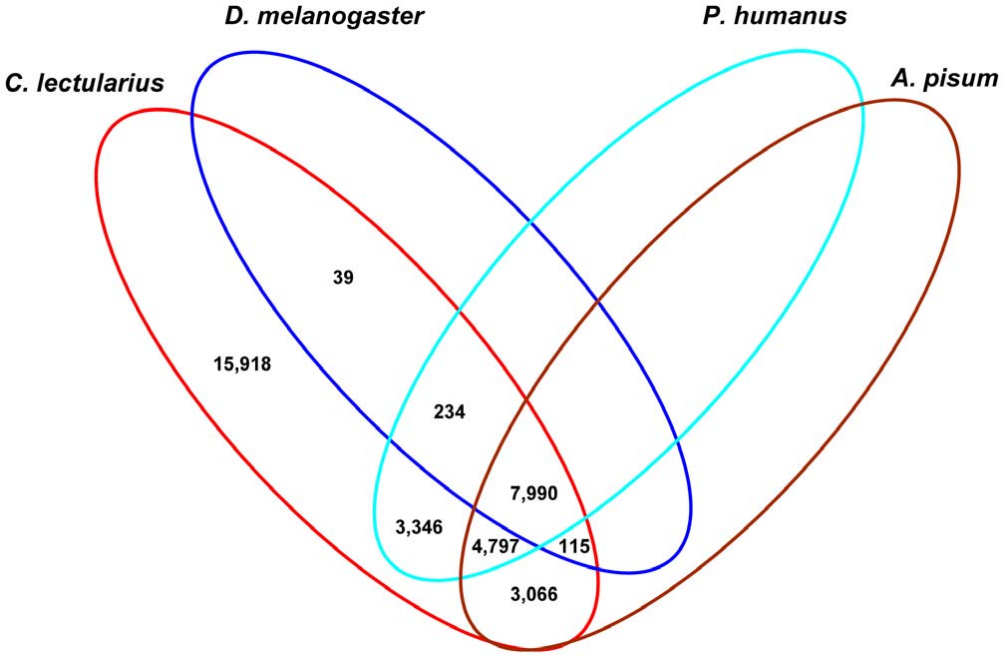
Comparative summary of *Cimex lectularius* transcriptomic sequences with the protein sequences of *Drosophila melanogaster, Pediculus humanus* and *Aphis pisum*.

The comparison of *C. lectularius* transcriptomic sequences to the draft protein sequences of three insect species [44], [45] revealed that the majority of sequences (46.1%, 16,367 of 35,505) were similar to *Pediculus humanus* (body louse) followed by *Acyrthosiphon pisum* (pea aphid), (45%) and *Drosophila melanogaster* (fruit fly) (23.6%) (Figure 3). High sequence similarity of *C. lectularius* with *P. humanus* might be due to their similar diet, i.e., blood. A significant percentage of transcripts (44.8%) were found to be unique to *C. lectularius* and perhaps could be attributed to the presence of novel genes. Alternatively, the derived transcripts may be from the cDNA of untranslated regions, chimerical sequences (assemblage errors) and non-conserved areas of proteins where homology is not detected, which is in agreement with several other transcriptomic studies [43], [46], [47].

### Gene Ontology assignments

**Figure 4:**
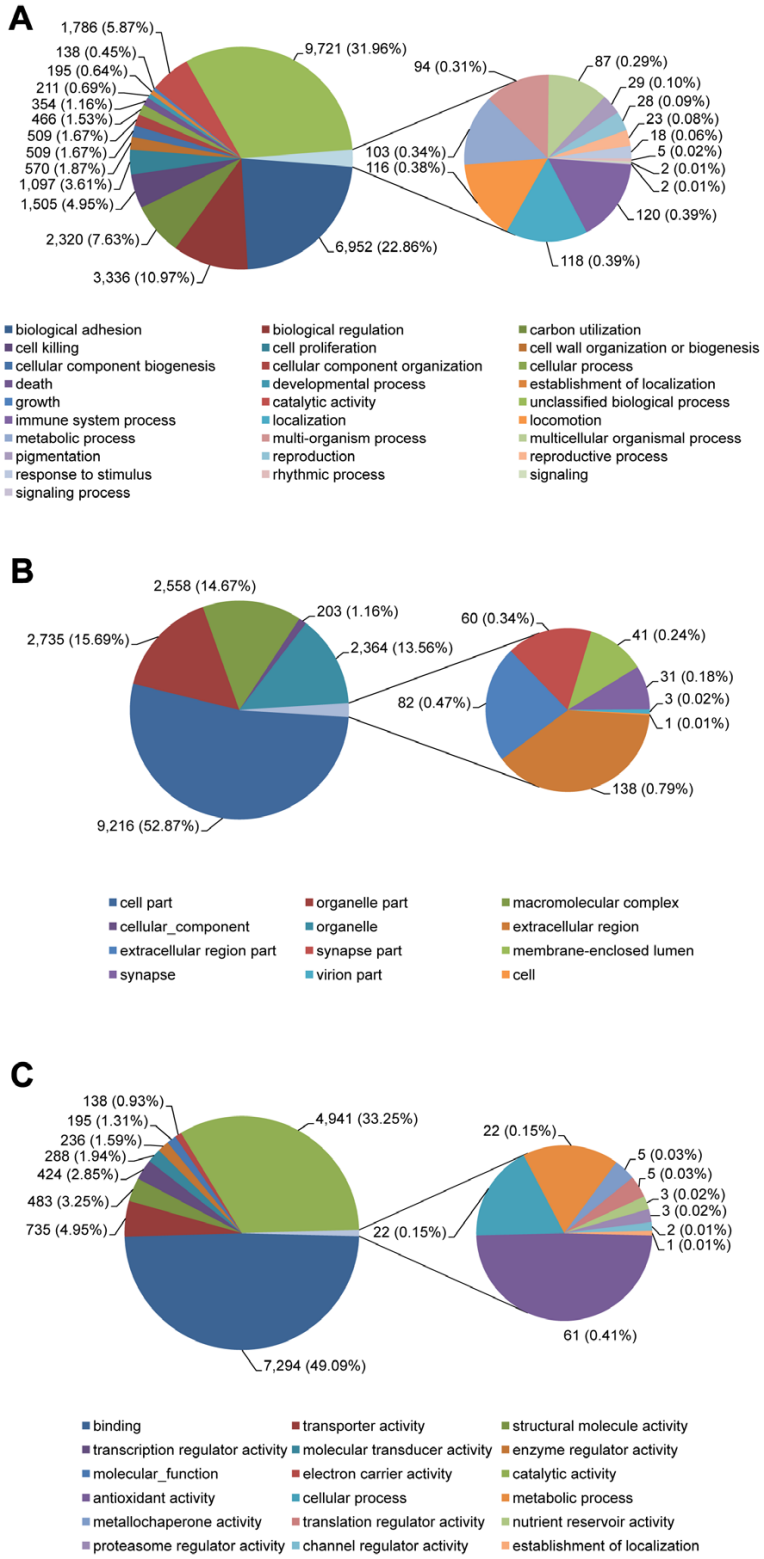
Gene ontology (GO) terms for the transcriptomic sequences of *Cimex lectularius.* (A) biological process, (B) cellular component and (C) molecular function.

In total 8,363 transcripts of *C. lectularius* were assigned for Gene Ontology (GO) terms based on BLAST matches with sequences whose function is previously known (Figure 4, Table S1). These transcripts were assigned for biological process (7,066 sequences, Figure 4a), cellular component (5,549 sequences, Figure 4b) and molecular function (6,290 sequences, Figure 4c). Among the molecular function assignments, a high percentage of genes were assigned for Binding (49.1%), predominantly heat shock proteins (Hsp). In a recent study of *C. lectularius*, the transcript levels for Hsp70 and Hsp 90 were observed to be elevated when bugs were subjected to various stress factors (heat, cold and dehydration) suggesting that these proteins may play an important role during environmental stress and could potentially play a role in control strategies [1], [8], [13], [48]. The cellular component terms showed a significant percentage of genes assigned to cell part (53%) whereas the biological process terms were associated predominantly with cellular processes (32%) such as proteolysis, carbohydrate metabolic processes and oxidation reduction utilization. Similar observations for metabolic processes were reported in transcriptomic studies of other insects [38], [43], [49].

### KEGG analysis

The KEGG metabolic pathways presented in the current EST database of *C. lectularius* were Nucleotide Metabolism (569 transcripts), Protein Metabolism (560), Lipid Metabolism (346), Alkaloid Metabolism (329), Carbohydrate Metabolism (295), Detoxification by cytochrome P450 (91), and Vitamin Metabolism (82) (Table S2). Taken together, the putative KEGG pathways identified in the current study shed light on specific responses and functions involved in the molecular processes of *C. lectularius*.

### Protein Domains

A total of 6,752 protein domains were identified in 6,286 *C. lectularius* transcripts using HMMER3 software (Table S3). Among these domains, lamprin proteins were the highest with a total of 223 (Table 1). Lamprin proteins are a unique family of hydrophobic self-aggregating proteins consisting of GGLGY tandem pentapeptide repeats reported in lamprey cartilage proteins, mammalian and avian elastins, and various insects (silk moth chorion protein and spider dragline silk) [50]–[53]. Protein kinase (82) and protein tyrosine kinase (55) were among the other top Pfam domains in our study. Both proteins are involved in signal transduction pathways, development, cell division and metabolism in higher organisms [54], [55]. Approximately 60 cytochrome P450 domains were predicted in the derived transcriptomic sequences of *C. lectularius*. Insect cytochrome P450s are reported in the metabolism of xenobiotics, wherein induced levels are correlated with resistance to synthetic insecticides and plant allelochemicals [56], [57].

**Table 1:** Summary of top 15 domains predicted in *Cimex lectularius* sequences.

Domain accession	Domain name	Domain description	Occurrence
PF06403.4	Lamprin	Lamprin	223
PF00069.18	Pkinase	Protein kinase domain	82
PF00067.15	p450	Cytochrome P450	60
PF00076.15	RRM_1	RNA recognition motif (a.k.a. RRM, RBD, or RNP domain)	58
PF07714.10	Pkinase_Tyr	Protein tyrosine kinase	55
PF00071.15	Ras	Ras family	54
PF00400.25	WD40	WD domain, G-beta repeat	53
PF00083.17	Sugar_tr	Sugar (and other) transporter	52
PF00153.20	Mito_carr	Mitochondrial carrier protein	47
PF00379.16	Chitin_bind_4	Insect cuticle protein	45
PF08477.6	Miro	Miro-like protein	39
PF07690.9	MFS_1	Major facilitator superfamily	38
PF00025.14	Arf	ADP-ribosylation factor family	35
PF07679.9	I-set	Immunoglobulin I-set domain	34
PF00118.17	Cpn60_TCP1	TCP-1/cpn60 chaperonin family	33

In total, 58 RNA recognition motifs (RRMs) were predicted in the *C. lectularius* sequences. These domains are also referred to as RNA-binding domain (RBD), consensus sequence RNA-binding domain (CS-RBD), ribonulceoprotein domain (RNPD), and RNP consensus sequence (RNP-CS). These proteins are involved in pre-mRNA processing and transport, regulation of stability and translational control [58], [59]. RRMs are reported to be involved in male courtship and vision in *D. melanogaster* [61], [62]. Mutations in *D. melanogaster* RRMs resulted in reduced viability, female sterility with abnormal wing and mechanosensory bristle morphology [58].

From the *C. lectularius* database we predicted 54 protein domains belonging to the Ras family, which are thought to be involved in insect development especially in cell differentiation and proliferation [62]. A high number of WD domains were identified in this study (Table 1), which are primarily involved in protein-protein interactions [36]. Sugar transporters (52) that are associated with transport of nutrients, and domains of mitochondrial carrier proteins (47), which are primarily involved in transport of metabolite intermediates, were also among the top ten domains predicted in the *C. lectularius* sequences [63], [64]. The later proteins are recognized by their unique signature motif P-X-[D/E]-X-X-[R/K] and the presence of six helical transmembrane segments made up of three tandem repeated sequences [65].

We predicted 45 insect cuticle proteins in the derived *C. lectularius* sequences. Insect cuticle is a complex structure consisting of chitin embedded in a protein matrix that lacks cysteine residues but is characterized with conserved R&R domain (G-x(7)-[DEN]-G-x(6)-[FY]-x-A-[DGN]-x(2,3)-G-[FY]-x-[AP] [66]–[68]. The R&R consensus is further classified into RR1 (soft cuticle), RR2 (hard cuticle) and extended R&R consensus chitin binding domain [66]. The other highly abundant domains identified in the present study include Miro-like protein (39), Major Facilitator Superfamily (38), ADP-ribosylation factor family (35), Immunoglobulin I-set domain (34) and TCP_1/cpn60 chaperonin family (33). Interestingly, we didn't find PAZ and PIWI domains, which are believed to be important components of the RNA induced silencing complex. The lack of these domains in our current database of *C. lectularius* could be attributed to insufficient coverage of the transcriptome.

### Genes of Interest

We have mined the current transcriptomic database to obtain genes putatively involved in insecticidal resistance of *C. lectularius* (Table 2). Given that one of the factors responsible for *C. lectularius* resurgence is purported to be pyrethroid resistance during the last decade, we are specifically interested in genes that participate in generalized insect defense. Metabolic resistance in insects has been attributed to induced levels of cytochrome P450 monoxygenases (CYPs), glutathione *S*-transferases (GSTs), superoxide dismutases (SODs), catalases (CATs), glutathione peroxidases (GPXs), carboxyl choline esterases and ascorbate peroxidases [69], [70]. Intriguingly, the majority of the cytochrome P450s identified in the *C. lectularius* transcriptome database belonged to the CYP3 clade (includes CYP3, CYP6 and CYP9 members) compared to other CYP clades, which is in agreement with other insect systems [56].

**Table 2:** Genes of interest in *Cimex lectularius*.

Candidate genes	#Occurrence	Family members with corresponding numbers
**Cytochrome P450**		
*Cyp2 clade*		
CYP18	01	CYPXVIIIA1
CYP307	03	CYPCCCVIIA1
CYP314	03	CYPCCCXIVA1
		
*CYP3 clade*		
CYP3	05	CYPIIIA1 (2), CYPIIIA12 (1), CYPIIIA13 (1), CYPIIIA31 (1)
CYP6	35	CYPVIA2 (1), CYPVIA13 (5), CYPVIA14 (9), CYPVIA18 (2), CYPVIA21 (3), CYPVIB2 (1), CYPVIB4 (1), CYPVID2 (2), CYPVID4 (1), CYPVID5 (4), CYPVIJ1 (4), CYPVIK1 (2)
CYP9	05	CYPIXE2
		
*CYP4 clade*		
CYP4	17	CYPIVC1 (5),CYPIVF8 (1),CYPIVG1 (2),CYPIVG15 (9)
		
*Mitochondrial CYP clade*		
CYP 301	04	CYPCCCIA1
		
**Glutathione S-transferases**	14	
**Superoxide dismutases**	06	
**Catalases**	02	
**Peroxidases (GPX and PhGPX)**	03	
**Voltage gated sodium channel**	01	
***Wolbachia***	59	

**Figure 5:**
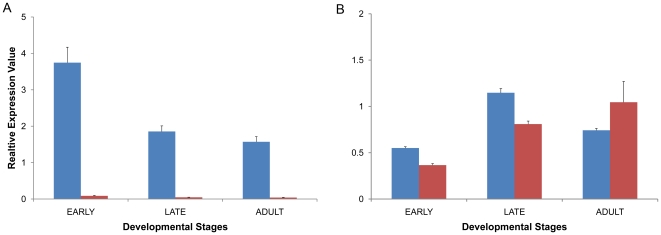
Quantitative PCR analysis of candidate defense genes in *Cimex lectularius* early- and late-instar nymphs and adults. (A) mRNA levels for a cytochrome P450 (CYP9) in pesticide-exposed (blue bars) and pesticide-susceptible populations (red bars). (B) mRNA levels for a glutathione-*S*-transferase (GST) in pesticide-exposed (blue bars) and pesticide-susceptible populations (red bars). An EF-1alpha was used as the internal reference gene. Standard error of the mean for three technical replicates is represented by the error bars.

Although pesticide resistance in *C. lectularius* is thought to be via point mutations in voltage-gated sodium channels [24], [26], the role of the detoxification and antioxidant enzymes is poorly understood. Hence, from the current database, we profiled the transcript levels for a cytochrome P450 (CYP9) and a GST (Delta-epsilon) in different developmental stages (early-instar nymphs, late-instar nymphs and adults) of pesticide-susceptible and pesticide-exposed *C. lectularius* populations. Quantitative real-time PCR (qPCR) analysis of the CYP9 showed higher mRNA levels for all developmental stages in pesticide-exposed populations compared to pesticide-susceptible populations (Figure 5A). In particular, the highest transcript levels for CYP9 were observed in early instars of the pesticide-exposed population. Similar observations were reported in *Heliothis viriscens* and *M. sexta* wherein CYP9A1 and CYP9A2 were over-expressed in response to insecticidal treatments [71], [72]. More recently, CYP9M10 of *Culex quinquefasciatus* was shown to be involved in pyrethroid detoxification [31]. Based on these studies, the CYP9 profiled in *C. lectularius* could also be induced upon pesticide exposure; however further functional studies (gene expression and RNAi) are required to elucidate the role of CYP9 in *C. lectularius*.

GSTs are thought to be potential secondary detoxification agents and are majorly involved in DDT resistance [73]. Expression analysis of a candidate GST retrieved from the current EST database revealed highest mRNA levels in the late-instar nymphs from pesticide-exposed *C. lectularius* populations compared to those of the pesticide-susceptible populations (Figure 5B). However, there was no significant difference in GST activity for adults from the two populations, an observation previously reported for adult bed bugs [26].

#### *Wolbachia*

In the current transcriptome database of *C. lectularius*, we found 59 sequences showing similarity with *Wolbachia* (Table 2, Table S4). As an endosymbiont, *Wolbachia* is reported in nearly 70% of all insect species [74]–[76]. Besides their role in nutrition, these are thought to play an important role in manipulating the host reproductive system through reproductive parasitism, i.e., feminization of genetic males, parthenogenesis and cytoplasmic incompatibilities, thereby increasing the frequency of infected females in the host population [77]–[82]. In a recent study, *Wolbachia* was shown to be essential for *C. lectularius'* synthesis of B vitamins, which are deficient in blood meals. Antibiotic-supplemented blood meals for *C. lectularius* resulted in delayed adult emergence and egg deposition; however, normal adult emergence and egg development was restored when the blood meal containing antibiotic was supplemented with B vitamins[82].

### Putative Molecular Markers

We predicted a total of 296 putative single nucleotide polymorphisms (SNPs) wherein 96 were transversions and 200 were transitions (Table 3, Table S5). Additionally, we identified 370 simple sequence repeats (SSRs or microsatellites), of which 69% were trinucleotide repeats, followed by 27% dinucleotide and 4% tetranucleotide repeats (Table 4, Table S6). Molecular markers identified in the current study could lay a platform for better understanding the adaptation/ecology of *C. lectularius* as reported in other insect systems [83]. However all the predicted molecular markers need to be validated to rule out false positives and sequencing errors.

**Table 3:** Predicted single nucleotide polymorphisms (SNP) in *Cimex lectularius* sequences.

SNP type	Counts
**Transition**	200
A-G	91
C-T	109
**Transversion**	96
A-C	21
A-T	31
C-G	16
G-T	28

**Table 4:** Summary of microsatellite loci predicted in *Cimex lectularius* sequences.

Number of repeats	Dinucleotide repeats	Trinucleotide repeats	Tetranucleotide repeats
5	-	151	12
6	-	58	01
7	-	21	02
8	41	10	-
9	26	04	-
10	10	03	-
11	06	02	-
12	02	01	-
13	03	-	-
14	-	03	-
15	02	01	-
16	03	01	-
17	03	-	-
20	01	-	-
21	01	-	-
22	01	-	-
35	01	-	-
Subtotal	100	255	15

### Conclusions

This study is the first to obtain fundamental molecular knowledge of *C. lectularius*. Some noteworthy results of this study are 1) a significant number of putative defense pathways were identified within the derived sequences; 2) a number of SNPs and microsatellite markers were predicted, which upon validation could facilitate the identification of polymorphisms within *C. lectularius* populations; and 3) high transcript levels for a cytochrome P450 (CYP9) in pesticide-exposed *C. lectularius* populations provide initial clues to metabolic resistance. These characteristic features along with the recovered sequences of *Wolbachia* provide new insights into the biology of *C. lectularius.*

## Materials and Methods

### Insect material

*C. lectularius* populations used in this study include the pesticide-susceptible strain “Harlan” which has been in laboratory culture since 1973 and hence has not experienced pesticide exposure for several decades. Pesticide-exposed bed bugs from Columbus, OH, were collected during 2009 and 2010 from an apartment that had undergone repeated insecticide treatments without successful bed bug control. These bed bug populations were reared in the laboratory as previous described [21]. Samples of the above-mentioned collections were transported to the Ohio Agricultural and Research Development Center (OARDC, Wooster, OH) and were categorized into different developmental stages as per Usinger [84].

### RNA isolation, cDNA library construction and 454 sequencing

Total RNA was extracted using TRIzol® reagent (Invitrogen) from a total of 15 individual insects of various developmental stages (1^st^-instar nymph– adult) of the Harlan strain. Approximately 10 µg of the extracted RNA was shipped to the Purdue Genomics Core Facility (West Lafayette, IN) for cDNA library construction and subsequent 454 sequencing. The cDNA library was constructed using the SMART cDNA library construction kit and following the manufacturer's protocol with a few modifications to enhance sequencing: i) a modified CDSIII/3′ primer (5′-TAG AGG CCG AGG CGG CCG ACA TGT TTT GTT TTT TTT TCT TTT TTT TTT VN-3′; PAGE purified) and SuperScript II reverse transcriptase (Invitrogen, Carlsberg, CA) were used for first-strand cDNA synthesis, and ii) cDNA size fractionation was excluded and final products were cleaned and eluted using a QIAquick PCR purification kit (Qiagen, Valencia, CA). Following agarose gel electrophoresis and extraction of DNA from gels, DNA bands (500-800 bp) were purified, blunt ended followed by ligation with adapters and finally immobilized on beads. Single stranded DNA isolated from the beads was characterized for correct size using a LabChip 7500. The concentration and the proper ligation of the adapters were examined using qPCR. One-quarter of a pico-titer plate was sequenced following manufacturer's protocol using the Roche 454 GS FLX Titanium chemistry (Roche Diagnostics, Indianapolis, IN).

### Bioinformatic analysis

The sequences were assembled using NEWBLER software package (a *de novo* sequence assembly software) after the removal of adapter sequences. For attaining better results, the contigs and singletons were renamed in the format of “BB454ONE000001” where “BB” stands for the bed bug species, “454” for 454 sequencing technology, “ONE” for the first trial, and “000001” for an arbitrarily assigned number. All the contigs and singletons of *C. lectularius* were analyzed using BLASTx algorithm [85] against GenBank non-redundant database at National Center for Biotechnology Information (NCBI) (http://www.ncbi.nlm.nih.gov/). Using BLASTx algorithm we also compared the sequences to the insect-specific protein sequences. To examine the protein domain all the sequences were searched against the Pfam database [86] by HMMER v3 program [87]. The Blast2GO software [88], [89] was used to predict the functions of the sequences, assign Gene Ontology terms, and predict the metabolic pathways in Kyoto Encyclopedia of Genes and Genome [90]–[92]. SSRs were identified using Msatfinder version 2.0.9 program [93] whereas SNPs were predicted using gsMapper software (Roche Diagnostics) with an arbitrary criterion of at least 4 reads supporting the consensus or variant.

### Gene mining and quantitative real time PCR

Total RNA was extracted from different development stages (early instars, late instars, and adults) using TRIzol® following the manufacturer's protocol. The total RNA obtained was re-suspended in 40 µl of nuclease-free water and the concentration was measured using Nanodrop (Thermo Scientific Nanodrop 2000). About 0.5 µg of total RNA was used as template to synthesize first-strand cDNA using Superscript II Reverse Transcriptase kit (Invitrogen) following the manufacturer's protocol. The resultant cDNA was diluted to 20 ng/µl for further use in qPCR. Genes of interest included CYP9 and a GST that were subjected to qPCR analysis. Primers were designed using Beacon Designer 7 software (primer sequences upon request). The cycling parameters were 95°C for 5 min followed by 40 cycles of 95°C for 10 s and 60°C for 30 s ending with a melting curve analysis (65°C to 95°C in increments of 0.5°C every 5 s) to check for nonspecific product amplification. Relative gene expression was analyzed by the 2^-ΔΔCT^ method (User Bulletin #2: ABI Prism 7700 Sequence Detection System vide supra (http://www3.appliedbiosystems.com/). An elongation factor 1-alpha (EF1-α) of *C. lectularius* was used as the internal reference gene, as has been used in other insect systems [94].

### Data deposition

The Roche 454 reads of *C. lectularius* were submitted to NCBI Sequence Read Archive under the accession number of SRA024509.1.

## Supporting Information

Table S1 Gene Ontology of *C. lectularius* sequences.
(XLSX)

Table S2 KEGG summary of *C. lectularius* sequences.
(XLSX)

Table S3 Pfam domain search of *C. lectularius* sequences.
(XLSX)

Table S4 Predicted *Wolbachia* sequences of *C. lectularius*.
(XLS)

Table S5 Putative SNPs in *C. lectularius* sequences.
(XLSX)

Table S6 Putative microsatellite loci in *C. lectularius* sequences.
(XLSX)
